# Excellent Outcomes With Stereotactic Body Radiotherapy in an Elderly Patient With Locally Progressive Immunotherapy-Resistant Renal Cell Carcinoma

**DOI:** 10.7759/cureus.85399

**Published:** 2025-06-05

**Authors:** Miriam Torrisi, Laura Giannini, Roberta Tummineri, Chiara Lucrezia Deantoni, Andrei Fodor

**Affiliations:** 1 Radiation Oncology, Istituto di Ricovero e Cura a Carattere Scientifico Ospedale San Raffaele, Milan, ITA

**Keywords:** carcinomas renal cell, clear cell renal carcinoma, cyber knife, robotic stereotactic radiotherapy, stereotactic radiotherapy (srt)

## Abstract

Clear cell renal cell carcinoma (ccRCC) is typically managed through surgical resection; however, many patients are not eligible for surgery due to advanced age or comorbidities. Stereotactic body radiotherapy (SBRT) has emerged as a promising noninvasive alternative, providing new opportunities for disease control in selected patients. We report the case of an 80-year-old male with metastatic ccRCC, initially treated with nivolumab plus ipilimumab according to the CheckMate 214 protocol. Due to an oligoprogressive renal lesion, SBRT was administered to a total dose of 40 Gy in five fractions (prescription isodose 80%) without significant toxicity. Immunotherapy was discontinued in June 2022 due to immune-related adverse events. Almost five years after SBRT, the treated lesions remain radiologically and clinically stable, despite the long-term discontinuation of systemic therapy. In this case, SBRT proved to be a valuable treatment option for a patient with RCC who was not a candidate for surgery or further systemic therapy. These findings suggest that integrating SBRT into the multimodal management of mRCC may provide prolonged disease control with minimal toxicity in carefully selected cases.

## Introduction

Renal cell carcinoma (RCC) accounts for approximately 80% of all malignant kidney tumors, making it the most common type of kidney cancer. Standard management for localized RCC typically involves surgical resection, such as partial or radical nephrectomy [1]. However, a substantial proportion of patients are not candidates for surgery due to factors such as tumor burden, comorbidities, advanced age, a solitary kidney, or poor performance status [2].

For these nonsurgical candidates, alternative treatments like thermal ablation often yield suboptimal disease control, particularly in tumors larger than 3 cm [3]. The effectiveness of thermal ablation is limited to small renal tumors situated away from the collecting system and major vascular structures due to the risks of heat sink effects, urinary strictures, or fistula formation. Additionally, attempting to treat larger tumors with these techniques increases the risk of hemorrhage, which may necessitate nephrectomy to control bleeding.

Traditionally, radiotherapy was used mainly for palliative care in RCC due to its perceived radioresistance under conventional fractionation schemes [4]. Consequently, systemic therapies, particularly targeted agents and immune checkpoint inhibitors (ICIs), have long been the primary treatment approach for metastatic RCC (mRCC). However, technological advancements in radiotherapy, especially stereotactic body radiotherapy (SBRT), have transformed this perspective. SBRT allows for the precise delivery of high-dose, hypofractionated radiation with steep dose gradients and submillimeter accuracy. It has shown promising local control (LC) rates in both primary and mRCC while minimizing toxicity to surrounding tissues [5,6]. Today, international guidelines recommend SBRT for selected patients with both primary tumors [5] and oligometastatic or oligoprogressive disease [6].

The rising interest in SBRT is supported by growing evidence from retrospective and prospective studies, which have demonstrated durable LC even in cases that were resistant to other local ablative therapies [5,7,8]. These findings are particularly encouraging in scenarios where systemic therapy alone may be insufficient or inappropriate.

Over the past decade, several targeted therapies have been approved for the treatment of mRCC [8-10]. More recently, the introduction of ICIs has significantly shifted the treatment paradigm. Dual checkpoint blockade with nivolumab and ipilimumab, as well as combinations of PD-L1 inhibitors with VEGFR tyrosine kinase inhibitors (TKIs), have demonstrated improved response rates, progression-free survival, and/or overall survival (OS) compared with sunitinib [11].

In this evolving treatment landscape, SBRT has emerged as a key option for managing oligometastatic disease, offering high LC rates, a favorable toxicity profile, and potential synergistic effects when combined with immunotherapy [11].

Here, we present a case of a patient with mRCC who was treated with SBRT for an oligoprogressive renal lesion.

## Case presentation

**Figure 1:**
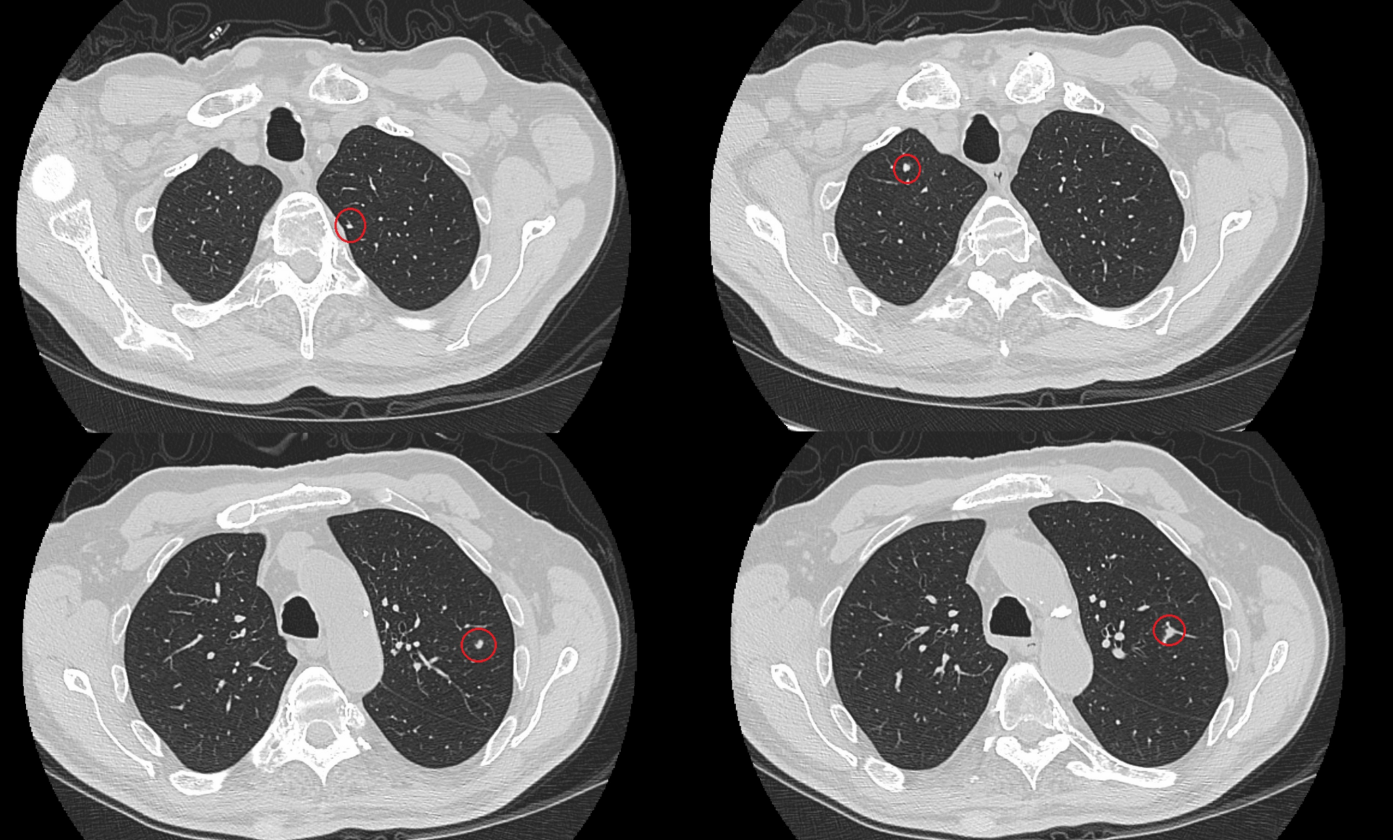
Axial chest CT scan showing pulmonary nodules highlighted in red

An 80-year-old male with a history of hypertension and mild chronic kidney disease was referred to our radiation oncology department after the incidental radiologic discovery of a suspicious renal mass during abdominal imaging performed for unrelated symptoms. In June 2019, contrast-enhanced CT revealed multiple bilateral renal lesions with imaging features suggestive of a primary renal neoplasm and contralateral metastasis. Histopathological confirmation via percutaneous biopsy, performed in September 2019, established the diagnosis of clear cell RCC. A staging chest CT identified multiple indeterminate pulmonary nodules suspicious for metastatic disease (Figure 1).

In December 2019, the patient began combination immunotherapy with nivolumab and ipilimumab, following the CheckMate 214 protocol. Due to treatment-related toxicity, ipilimumab was discontinued in May 2020 after the onset of immune-mediated pancreatitis, while nivolumab monotherapy was continued.

In January 2021, a progressive renal lesion was identified, showing an increase in size of 3 × 6 mm. Given the patient’s comorbidities and oligoprogressive status, SBRT was proposed as a definitive local treatment.

Treatment phase

SBRT was delivered to the left renal lesion using a CyberKnife M6 system with real-time tracking (Synchrony; Accuray, Inc., Sunnyvale, California, USA). This technique involved implanting four fiducials in or near the tumor under ultrasound guidance, which acted as radiographic markers for the Synchrony tracking system. Three fiducials were placed within the lesion, and one was positioned in the space between the lesion and the left psoas muscle.

One week after implantation, a planning contrast-enhanced CT scan with 1.25-mm slice thickness was performed, with the patient lying supine, arms at his sides, and ankles and knees secured with a Combifix™ (CIVCO Medical Solutions, Coralville, Iowa, USA).

**Figure 2:**
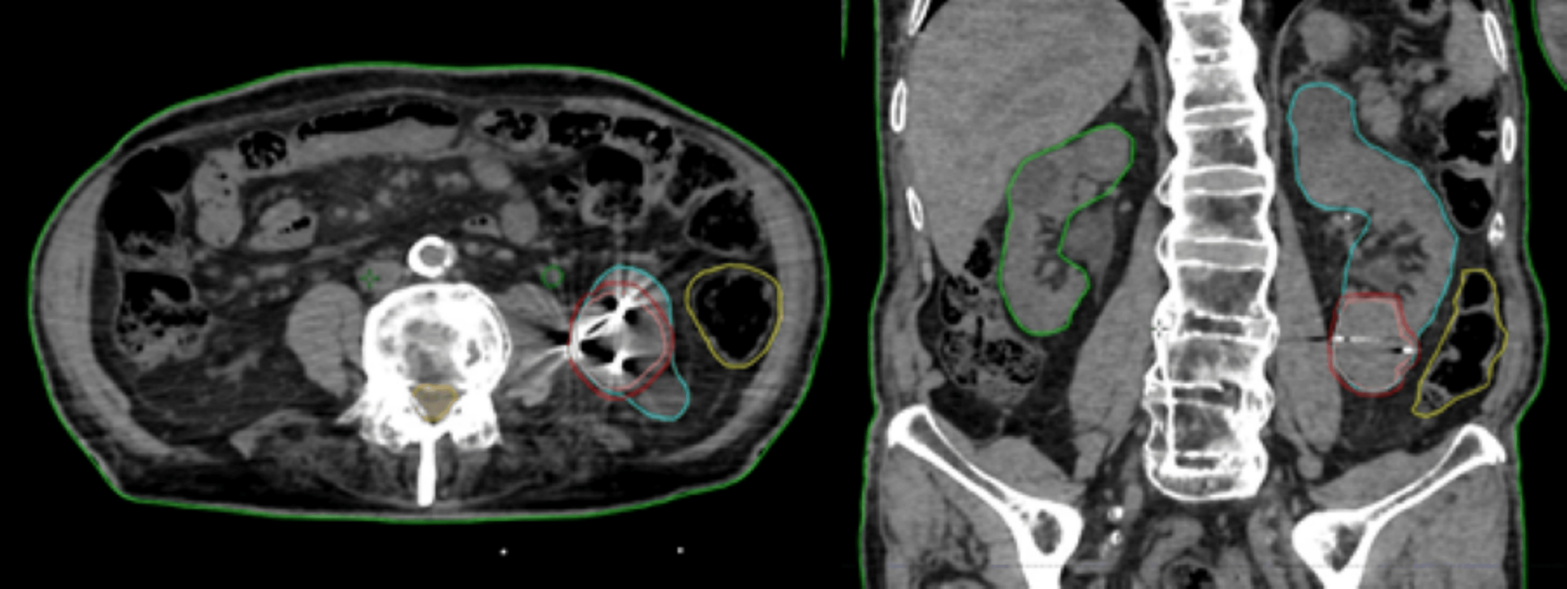
Left renal volume delineation from the treatment planning CT scan in axial (left) and coronal (right) planes

The gross tumor volume (GTV), defined as the radiographically visible tumor on CT images, and organs at risk, including both kidneys, bowel, renal hilum, and spinal cord, were delineated. The planning target volume was generated by adding a 3-mm margin around the GTV, with no additional margin for clinical target volume (Figure 2).

Treatment planning was performed using Accuray’s Precision Treatment Planning Multiplan system, version 2.0.1.1 (Accuray, Inc.).

**Figure 3:**
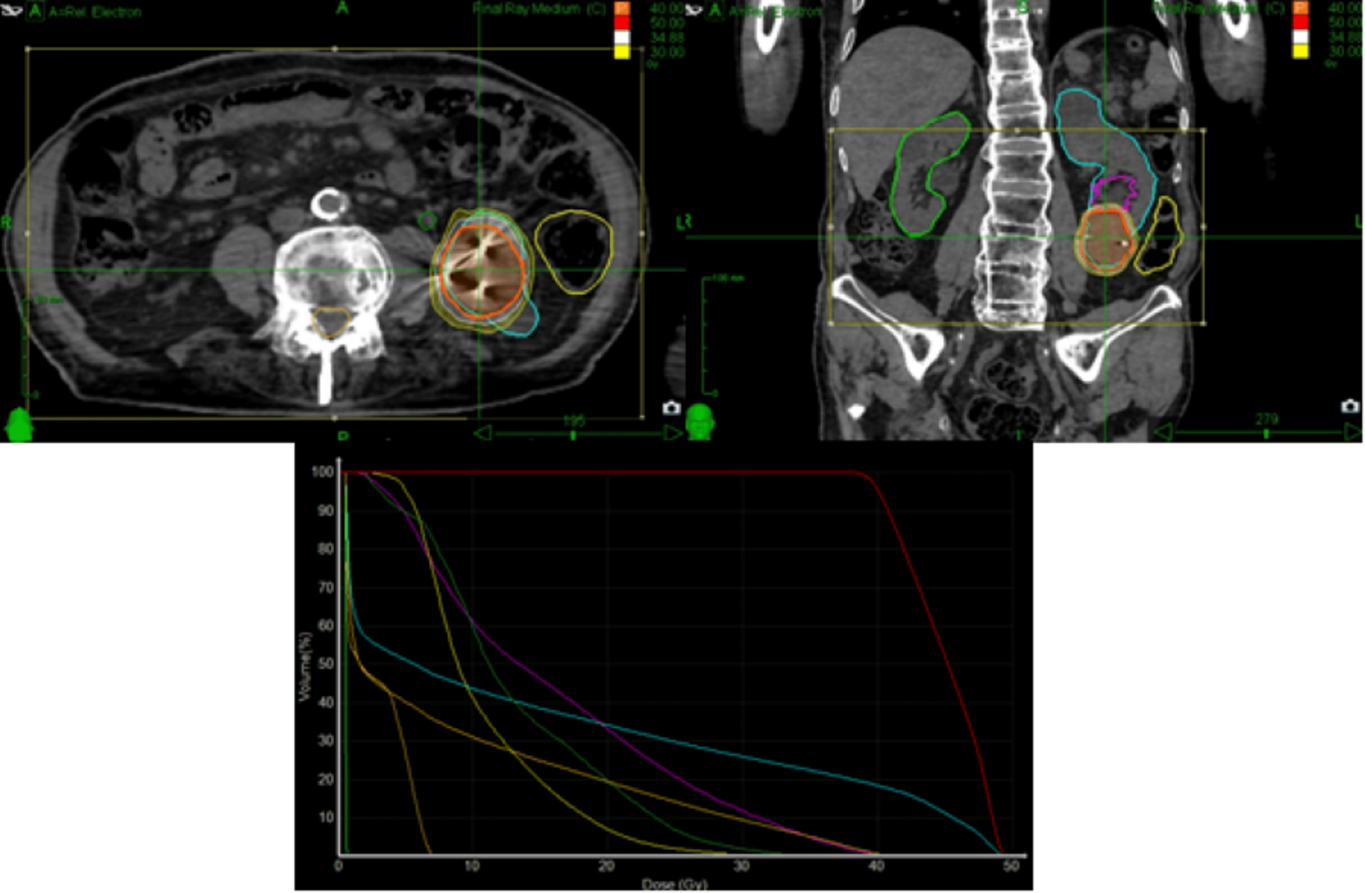
Axial and coronal views from the patient’s SBRT treatment plan illustrating the radiation dose distribution SBRT, stereotactic body radiotherapy

SBRT was delivered to the left renal lesion with a total dose of 40 Gy, administered in five consecutive fractions over one week (prescription isodose 80%). The dose distribution and dose-volume histogram are shown in Figure 3. Dose-volume constraints for critical organs and dose coverage for target volumes are summarized in Table 1 and Table 2.

**Table 1:** Dose-volume constraints for critical organs

Organ	Endpoint	Constraint	Dmax (0.03 cc) (Gy)
Colon	Colitis/fistula	V25Gy < 20 cc	38
Renal hilum/vascular trunk	Malignant hypertension	V23Gy < 66%	/
Renal cortex	Basic renal function	D200cc < 17.5 Gy	/
Spinal cord	Mielite	V23Gy < 0.5 cc; V14.5Gy < 1.2 cc; V23Gy < 10%; D1cc < 21 Gy	30
Stomach	Ulceration/fistula	V18 < 10 cc	32
Jejunum/ileum	Enteritis/obstruction	V19.5 Gy < 5 cc	35
Liver	Basic liver function	D700cc < 21 Gy	

**Table 2:** Dose coverage for the target volume GTV, gross tumor volume; PTV, planning target volume

Volume	Dmax	Dmin	Dmean	D98%	CI
PTV	50 Gy	36.85 Gy	44.94 Gy	39.46 Gy	1.03
GTV	50 Gy	41.02 Gy	46.65 Gy	42.78 Gy	1.52

SBRT combined with immunotherapy was well tolerated, with no acute toxicities of grade 2 or higher reported.

Follow-up phase

A follow-up schedule was established with blood tests and contrast-enhanced body MRI/CT every three months during the first year and every six months thereafter.

Three months after treatment, follow-up contrast-enhanced MRI showed a reduction in lesion size from 38 mm to 30 mm. The patient reported no treatment-related symptoms, and serum creatinine levels remained stable compared to pre-SBRT values, indicating preserved renal function.

In June 2022, the patient developed a grade 2 cutaneous rash and experienced a decline in renal function, with creatinine rising to a peak of 2.5 mg/dL, raising concern for immune-related nephritis. Consequently, nivolumab was permanently discontinued, and the patient was placed under close clinical and radiological follow-up.

At the last follow-up, 42 months after SBRT, the patient remained in good clinical condition with stable overall disease status.

## Discussion

RCC has traditionally been regarded as radioresistant, which has limited the role of radiotherapy primarily to palliative care. However, the advent of SBRT has challenged this view. Multiple studies have shown that SBRT can achieve durable LC in both primary and mRCC, reporting high LC rates (up to 100%) with minimal toxicity [7,12,13].

Preclinical research supports a strong radiobiological rationale for SBRT in RCC. The low alpha/beta ratio observed in RCC cell lines suggests a relative sensitivity to high-dose-per-fraction regimens [14]. Additionally, SBRT promotes immunogenic cell death, enhances major histocompatibility complex class I expression, and stimulates antigen presentation, potentially boosting systemic antitumor immune responses [15].

The combination of nivolumab and ipilimumab was recently approved for intermediate- or poor-risk mRCC based on the CheckMate 214 study [8]. With a minimum follow-up of four years, this combination demonstrated superior overall and complete response rates compared to sunitinib. Regarding safety, the regimen was associated with fewer severe adverse events overall but showed a higher rate of treatment discontinuation due to immune-related toxicities.

Integrating SBRT with immunotherapy is an emerging approach, especially relevant in the era of ICIs. The CheckMate 214 trial established nivolumab plus ipilimumab as a first-line standard for intermediate- and poor-risk advanced RCC, demonstrating superior OS and objective response rates relative to sunitinib [8].

Although clinical data on combining SBRT and ICIs in mRCC are still limited, the findings are encouraging. The phase II NIVES study assessed nivolumab plus SBRT in pretreated mRCC patients, showing good safety and local response rates, albeit without significant improvement in overall response rate compared to historical controls [11]. Similarly, a recent AIRO multicenter study confirmed that SBRT achieves high LC rates (>80% at 2 years) without increased toxicity when given alongside systemic treatments like nivolumab or TKIs [13]. A systematic review by Ingrosso et al. also reported that combining SBRT with systemic drugs in mRCC is safe and associated with favorable oncologic outcomes [16].

The TROG 15.03 FASTRACK II trial [13], the first multicenter prospective phase II study on stereotactic ablative body radiotherapy for primary RCC, enrolled 70 inoperable patients with biopsy-confirmed T1-T2 tumors. Despite a median tumor size of 4.6 cm and a significant comorbidity burden (mean eGFR: 61.1 mL/min/1.73m²), the trial reported 100% LC and cancer-specific survival at 12 months, with no treatment-related deaths and a manageable toxicity profile. Renal function showed a slight decline post-treatment but stabilized after 24 months, suggesting long-term preservation in most patients.

One of the most intriguing topics is the effect of local SBRT on distant disease control [17]. The “abscopal effect,” where localized radiation induces systemic tumor responses, has been documented in RCC and other cancers, especially when radiotherapy is combined with systemic therapy. Although these effects remain anecdotal, they suggest an important immunomodulatory role for SBRT [18,19].

Our case, showing durable disease control despite discontinuation of systemic therapy, supports these observations. SBRT may have acted not only as a local treatment but also as a consolidative or immunologic enhancer in a previously immune-primed patient.

Prospective randomized trials are needed to better define the optimal sequencing, patient selection, and potential synergy between SBRT and systemic treatments in RCC. Until then, careful patient selection, as demonstrated here, supports the use of SBRT as a safe and effective option within the broader treatment landscape of RCC.

## Conclusions

This case highlights the potential feasibility and safety of SBRT as a local treatment option for RCC in patients with mRCC. The durable disease control observed despite discontinuation of immunotherapy suggests that SBRT may have a valuable role in selected clinical scenarios.
